# A Tight Control of Non-Canonical TGF-β Pathways and MicroRNAs Downregulates Nephronectin in Podocytes

**DOI:** 10.3390/cells11010149

**Published:** 2022-01-03

**Authors:** Nina Sopel, Alexandra Ohs, Mario Schiffer, Janina Müller-Deile

**Affiliations:** 1 Department of Medicine 4–Nephrology and Hypertension, Universitätsklinikum Erlangen, Friedrich-Alexander-Universität Erlangen-Nürnberg, 91054 Erlangen, Germany; nina.sopel@uk-erlangen.de (N.S.); alexandra.ohs@uk-erlangen.de (A.O.); mario.schiffer@uk-erlangen.de (M.S.); 2 Research Center On Rare Kidney Diseases (RECORD), Universitätsklinikum Erlangen, 91054 Erlangen, Germany

**Keywords:** podocytes, nephronectin, TGF-β, podocytopathies, post-transcriptional regulation, microRNAs

## Abstract

Nephronectin (NPNT) is an extracellular matrix protein in the glomerular basement membrane that is produced by podocytes and is important for the integrity of the glomerular filtration barrier. Upregulated transforming growth factor β (TGF-β) and altered NPNT are seen in different glomerular diseases. TGF-β downregulates NPNT and upregulates NPNT-targeting microRNAs (miRs). However, the pathways involved were previously unknown. By using selective inhibitors of the canonical, SMAD-dependent, and non-canonical TGF-β pathways, we investigated NPNT transcription, translation, secretion, and regulation through miRs in podocytes. TGF-β decreased NPNT mRNA and protein in cultured human podocytes. TGF-β-dependent regulation of NPNT was meditated through intracellular signaling pathways. Under baseline conditions, non-canonical pathways predominantly regulated NPNT post-transcriptionally. Podocyte NPNT secretion, however, was not dependent on canonical or non-canonical TGF-β pathways. The canonical TGF-β pathway was also dispensable for NPNT regulation after TGF-β stimulation, as TGF-β was still able to downregulate NPNT in the presence of SMAD inhibitors. In contrast, in the presence of different non-canonical pathway inhibitors, TGF-β stimulation did not further decrease NPNT expression. Moreover, distinct non-canonical TGF-β pathways mediated TGF-β-induced upregulation of NPNT-targeting miR-378a-3p. Thus, we conclude that post-transcriptional fine-tuning of NPNT expression in podocytes is mediated predominantly through non-canonical TGF-β pathways.

## 1. Introduction

The glomerular filtration barrier (GFB) is the highly specialized interface within the glomerulus where the primary filtrate is generated. It is composed of fenestrated glomerular endothelial cells, the glomerular basement membrane (GBM), and podocytes. In addition, a glycocalyx composed of glycoproteins, including proteoglycans, coats the luminal surface of the glomerular capillaries, as well as the podocyte foot processes facing the urinary space [1,2].

Glomerular endothelial cells and podocytes contribute to the regulation of extracellular matrix (ECM) synthesis and organization in the GBM [3]. Genetic mutations in GBM components such as collagen IV α3α4α5 or laminin highlight the importance of glomerular ECM composition for normal glomerular barrier function [4]. Mutations that affect collagen IV α3α4α5 components cause Alport syndrome or thin basement membrane nephropathy, which presents with hematuria and can progress to end stage renal disease. Impairments in the synthesis or function of laminin α5β2γ1 lead to Pierson syndrome, characterized by congenital nephrotic syndrome with ocular abnormalities and neurological dysfunction [5].

Recently, microRNAs (miRs) have been described to regulate several cellular processes including the formation, maintenance, and remodeling of the ECM [6]. miRs are a family of small non-coding RNA molecules, consisting of 18–25 nucleotides, which post-transcriptionally regulate gene expression. Multiple miRs are regulated by transforming growth factor beta (TGF-β) in different organs, including the kidney [7,8,9].

In addition to collagens and laminins, nephronectin (NPNT) is another ECM protein of the GBM. We previously described that podocyte-derived miR-378a-3p and endothelial-cell-derived miR-192-5p downregulate NPNT in podocytes [10,11]. Furthermore, TGF-β upregulated miR-378a-3p and downregulated NPNT, suggesting a TGF-β-mediated miR-378a-3p–NPNT axis [10,12]. Knockdown of npnt/Npnt expression by a miR-378a-3p mimic induced proteinuria, due to impaired integrity of the GFB, with a thickening of the GMB and podocyte effacement in zebrafish and mice [10]. In human membranous glomerulonephritis, miR-378a-3p was upregulated in urine and in glomeruli, whereas NPNT was downregulated, confirming the importance of the TGF-β–miR-378a-3p–NPNT axis in renal pathology. However, miR-378a-3p inhibitor experiments in cultured human podocytes suggest that TGF-β also regulates NPNT independently of miRs [10]. Thus, the TGF-β-dependent regulation of NPNT in the podocyte remains to be discovered in more detail.

**Figure 1:**
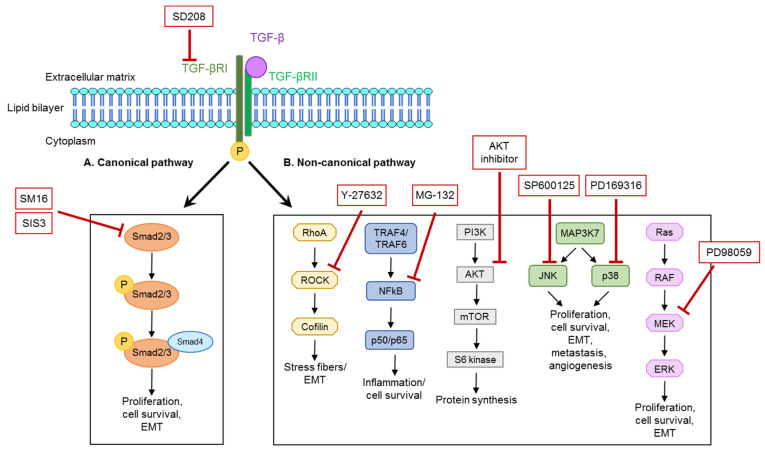
Illustration of canonical and non-canonical TGF-β pathways and the respective inhibitors used for the blockade of separate parts of the pathways. TGF-β signaling is intracellularly mediated via a canonical, SMAD2- and SMAD3-dependent pathway or different non-canonical pathways, which signal via different routes, namely the Rho-ROCK, NFkB, PI3K-AKT-mTOR, and MAP-kinase pathways via JNK or p53 and Ras-Raf-MEK-ERK. Each signaling arm facilitates different reactions in the cell, such as proliferation, cell survival, epithelial-to-mesenchymal transition (EMT), or protein synthesis (adapted from [18]).

Upon ligand stimulation, TGF-β receptor complexes are known to activate multiple intracellular signaling pathways, including SMAD-dependent (canonical pathway) and different non-canonical pathways, where signals are mediated via RhoA/Rock, TRAF-NFκB p, PI3K/Akt, MAP kinases (e.g., JNK and p38), and Ras/RAF (Figure 1) [13,14]. The diverse TGF-β pathways are known to cause different cellular effects. In podocytes, SMAD3 is required for the activation of pro-apoptotic caspase 3, whereas Cd2ap-dependent activation of PI3K/Akt mediates cell survival signaling [15,16]. In canonical TGF-β signaling, SMAD proteins translocate to the nucleus and regulate the transcription of target genes. SMADs also form a complex with chromatin remodeling proteins and promote the formation of active chromatin [17].

TGF-β signaling is able to adapt to particular environmental circumstances. In this regard, feedforward, as well as negative and positive feedback loops, are widely exploited to fine tune signal transduction [19].

In our previous study, we observed that the knockdown of NPNT caused podocyte effacement, whereas glomerular endothelial cells were not affected [10,11]. Transgenic mice with increased plasma levels of TGF-β developed glomerular disease [20]. These data suggest that chronically elevated TGF-β is a driving force of progressive renal disease. We therefore analyzed the long-term rather than short-term effects of elevated TGF-β on podocyte NPNT. We discovered that different non-canonical pathways regulate NPNT protein expression in podocytes but hardly influence NPNT mRNA expression. Furthermore, podocyte secretion of NPNT was independent from TGF-β pathways, hinting at post-transcriptional regulation. Indeed, NPNT-targeting miR-378a-3p was upregulated after TGF-β stimulation and was dependent on distinct non-canonical TGF-β pathways.

As TGF-β and NPNT are dysregulated in different glomerular diseases [10,11,12,13,14,15,16,17,19,20,21,22,23], our results of a TGF-β-mediated NPNT regulation, facilitated mainly by non-canonical pathways and post-transcriptional mechanisms, might have important impact for the development of novel therapeutic strategies in the future.

## 2. Materials and Methods

### 2.1. Cell Culture

In this study, conditionally immortalized human podocytes were used, which were initially created by Saleem et al. [24] and now represent a well-established cell culture model in podocyte research around the world. This podocyte cell line proliferates under permissive conditions at 33 °C. When cultivated at 37 °C, the SV40 T-antigen is inactivated for cell differentiation. The culture medium for human podocytes was RPMI 1640 Medium (Gibco, via Thermo Fisher Scientific, Waltham, MA, USA) with 10% fetal calf serum (FCS; PAN-Biotech, Aidenbach, Germany), 1% Penicillin/Streptomycin, and 0.1% Insulin-Transferrin-Selenium (Gibco).

To further investigate TGF-β signaling via the canonical pathway, we used conditionally immortalized murine Smad2 and Smad3 double-deficient (Smad2/3^-/-^) podocytes, which were a kind gift from Prof. Dr. Ilse Daehn, Icahn School of Medicine at Mount Sinai, New York, NY, and wild type (WT) control murine podocytes [25]. In contrast to conditionally immortalized human podocytes, immortalized murine podocytes were cultured on type I collagen-coated flasks (Corning, NY, USA). Furthermore, to enhance the expression of the thermosensitive T antigen, murine podocytes were cultured in the presence of 10 U/mL Interferon gamma (IFNγ) at 33 °C. For differentiation at 37 °C, the expression of the thermosensitive T antigen is inactivated. Therefore, during differentiation, no IFNγ is added to the culture medium. The murine cell culture medium was RPMI 1640 (Gibco) supplemented with 10% FCS (PAN-Biotech) and 1% Penicillin/Streptomycin.

For experiments, cells were differentiated for 10 to 12 days. In human immortalized podocytes, the inhibition of global, canonical, as well as non-canonical TGF-β signaling was carried out with specific inhibitors (Figure 1) in the following concentrations: SD208 (20 µM), SM16 (20 µM), SIS3 (5 µM), PD169316 (10 µM), SP600125 (10 µM), PD98059 (20 µM), AKT inhibitor (5 µM), MG-132 (0.5 µM), and Y-27632 (1 mM). After starvation in human podocyte media containing 1% FCS, cells were pre-incubated for 30 min with the inhibitors, followed by incubation for 16 to 24 h in normal culture medium in the presence or absence of TGF-β (5 ng/mL).

Murine Smad2/3^-/-^ and WT podocytes were treated with or without TGF-β (5 ng/mL) in murine podocyte media for 24 h.

Cell viability and morphology were determined on an optical microscope before and after every treatment.

### 2.2. RNA Isolation, cDNA Transcription, and qPCR

RNA from whole cell lysates was isolated using the ReliaPrep™ RNA Miniprep System (Promega, Madison, WI, USA), according to the manufacturers’ protocol. For mRNA reverse transcription, 1μg of RNA was transcribed using the following reagents: 5× RT buffer and M-MLV RT 50.000 U (Promega), dNTP Mix (Promega), random hexamer primer (ThermoFisher Scientific), and RiboLock (Thermo Fisher Scientific). Reverse transcription was carried out at 25 °C for 5 min, 40 °C for 60 min, and 70 °C for 10 min. Sybr green-based real-time PCR (Maxima SYBR Green/ROX qPCR Master Mix, Thermo Fisher Scientific) was performed with the following protocol: 10 min at 95 °C followed by 40 cycles of 15 s at 95 °C and 1 min at 60 °C, followed by 15 s at 95 °C, 1 min at 60 °C and 15 s at 95 °C. Individual samples were run in triplicates. The following primers were used: hNPNT fw: GGAGGCAAACACAGATCACC, hNPNT rev: TCCAATCTCCCCAGTGTGAC, hHPRT fw: GACCAGTCAACAGGGGACAT, and hHPRT rev: AACACTTCGTGGGGTCCTTTTC. Data was analyzed by using the ΔΔCt method.

### 2.3. MicroRNA Isolation, cDNA Transcription, and TaqMan PCR

The isolation of microRNAs was performed by using the miRNeasy Mini Kit (Qiagen, Hilden, Germany), and cDNA transcription and TaqMan miR Assays were done with TaqMan MicroRNA Assays for hsa-miR-378a-3p and U6 control miR (Thermo Fisher Scientific).

### 2.4. Western Blot

Cells were lysed in RIPA buffer containing protease and phosphatase inhibitors. Twenty μg of podocyte cell lysate were resolved in 10% SDS-PAGE and transferred to a nitrocellulose membrane by wet blot transfer. The detection of protein bands was performed using horseradish-peroxidase-labelled secondary antibodies and visualized using enhanced chemiluminescence reagents. The primary antibodies used: mouse-anti POEM (G-1, Santa Cruz, Dallas, TX, USA), mouse-anti GAPDH (6C5, Santa Cruz), rabbit-anti bTubulin (9F3, Cell Signaling), rabbit-anti Giα3 (06-270, Sigma Aldrich, St. Loius, MO, USA), rabbit-anti Lamin A/C (#2032, Cell Signaling, Danvers, MA, USA), and mouse-anti β-Actin (sc-47778, Santa Cruz). The secondary antibodies used: anti-mouse HRP and anti-rabbit HRP (DAKO, Agilent, Santa Clara, CA, USA).

A semi-quantitative analysis of protein expression was performed using ImageJ software (National Institutes of Health, Bethesda, MD, USA) by measuring band intensities of target proteins and a reference protein, as indicated. Fold expression was calculated.

### 2.5. Cell Fractionation

Cultured podocytes were harvested after 10 days of differentiation with trypsin/EDTA. The cell pellet was subsequently treated with buffers from the Subcellular Protein Fractionation Kit for Cultured Cells (Thermo Fisher Scientific), as recommended by the manufacturer, to obtain cytosolic, nuclear, and membrane fractions.

### 2.6. NPNT Elisa

Measurement of the NPNT protein concentration in the cell culture supernatant of podocytes was performed with the human Nephronectin ELISA kit (MyBioSource, San Diego, CA, USA), according to the manufacturers’ protocol.

### 2.7. CHX Chase Assay

For protein turnover experiments, differentiated podocytes were cultured in the presence of 10 µM cycloheximide (Sigma Aldrich) for the indicated time-points.

### 2.8. Data Analysis/Statistics

All data are shown as means ± SEM and were compared by ANOVAs or Student’s t-tests to test for statistical significance. The data were normally distributed.

## 3. Results

### 3.1. Podocyte NPNT Is Downregulated after Stimulation with TGF-β

We could previously show that podocytes express NPNT mRNA and protein [10,11]. Cell fractionation of human podocytes could only detect intracellular NPNT protein in the cytosolic fraction but not in the membrane or nucleus (Figure S1a). We also investigated the intracellular NPNT protein half-life in human podocytes. When protein biosynthesis is inhibited by cycloheximide (CHX), which prevents translational elongation, it is possible to estimate the protein turnover rate under steady state conditions. Cycloheximide chase assay determined NPNT was a short-lived protein when de novo protein synthesis was blocked (Figure S1b), hinting at secretion after protein synthesis, which is in line with it being deposited in the ECM [26].

**Figure 2:**
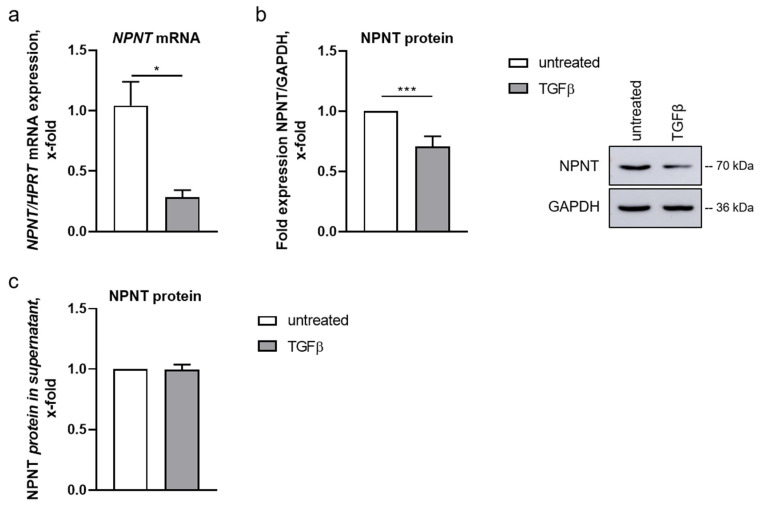
NPNT expression and localization in human podocytes. (**a**,**b**): *NPNT* mRNA (**a**) and protein (**b**) expression in cultured human podocytes, untreated and after TGF-β treatment. NPNT expression was normalized to *HPRT* and GAPDH, respectively, and is given as change compared to untreated cells. A representative Western blot picture is shown. *n* = 3–17 independent experiments, * *p* < 0.05, *** *p* < 0.001. (**c**): Change in NPNT protein concentration measured in the cell culture supernatant of untreated cultured human podocytes and after stimulation with TGF-β. *n* = 5 independent experiments.

Treatment with TGF-β for 16 or 24 h decreased NPNT mRNA and protein levels in human podocytes, respectively (Figure 2a,b). NPNT is an extracellular protein that is deposited in the glomerular basement membrane. Thus, a reduction in NPNT protein after TGF-β stimulation could have been due to an increase in NPNT secretion. Therefore, we analyzed NPNT protein concentration in the cell culture supernatant of human podocytes after stimulation with TGF-β. While we were able to detect NPNT protein in the podocyte cell culture supernatant, both with and without TGF-β treatment, no significant difference was discovered (Figure 2c). Furthermore, TGF-β did not significantly influence the NPNT protein half-life in the presence of CHX (Figure S1b).

### 3.2. TGF-β-Dependent Regulation of NPNT Protein Is Mediated through Intracellular Signaling Pathways

**Figure 3:**
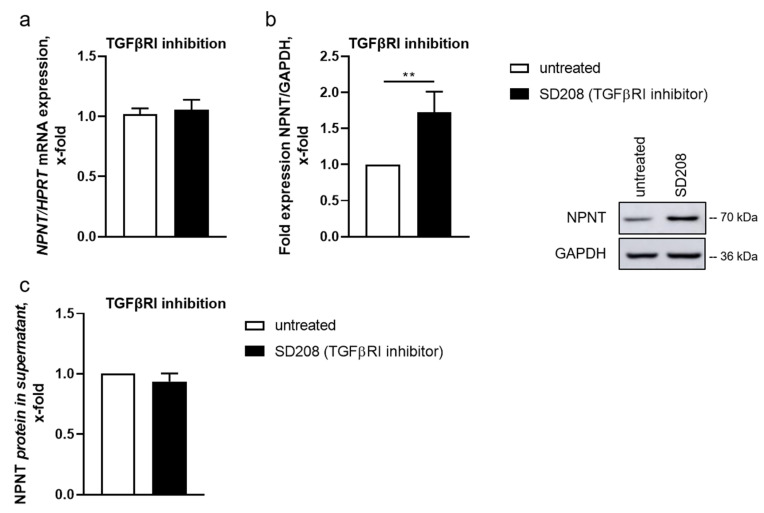
TGF-β-induced downregulation of NPNT is mediated by TGF-βR1. (**a**,**b**): qPCR (**a**) and Western blot (**b**) for NPNT mRNA and protein expression, respectively, in cultured human podocytes, untreated and after TGF-βR1 inhibition with SD208. NPNT expression was normalized to *HPRT* and GAPDH, respectively, and is given as the change compared to untreated cells. A representative Western blot picture is shown. *n* = 6–8 independent experiments, ** *p* < 0.01. (**c**): NPNT protein concentration in cell culture supernatant of human podocytes, untreated and after treatment with SD208 (TGF-βR1 inhibitor), given as the change compared to untreated cells. *n* = 4 independent experiments.

TGF-β binds to a TGF-β receptor type II dimer on the cell surface, which recruits a type I receptor (TGF-βR1) dimer, forming a hetero-tetrameric complex with the ligand [27]. To inhibit intracellular TGF-β signaling, we incubated human podocytes with a TGF-βR1 inhibitor (SD208). SD208 is an ATP-competitive TGF-βR1 inhibitor without activity against other related kinases [18,28]. In the presence of SD208, NPNT mRNA levels were comparable to control cells (Figure 3a). However, NPNT protein expression increased significantly after inhibition of TGF-βR1 (Figure 3b), while inhibiting the receptor did not affect NPNT secretion (Figure 3c), indicating that NPNT is predominantly regulated post-transcriptionally under baseline conditions.

### 3.3. Non-Canonical Pathways Predominantly Mediate Regulation of NPNT under Baseline Conditions

TGF-β mediates its downstream effects through canonical and non-canonical pathways [15,16]. The canonical pathway acts through the phosphorylation of SMAD2 and SMAD3 (SMAD-dependent pathway). The non-canonical signaling is mediated via different pathway routes that include RhoA/ROCK, NFκB, PI3K/AKT, JNK/p38, and the Ras-dependent MEK pathways [29].

First, we investigated how downstream TGF-β pathways regulate NPNT expression at baseline by treating human podocytes with different TGF-β pathway inhibitors. To impede the canonical pathway, we used the compounds SM16 and SIS3, which inhibit SMAD2 and SMAD3 phosphorylation [30]. For targeting the several arms of the non-canonical pathways, we used different inhibitors. PD169316 was used to inhibit the p38 pathway. Low concentrations of PD169316 are known to inhibit the activation of p38 kinase by 40% but do not affect JNK activity [31]. Higher concentrations of PD169316, which we used in our experiments, inhibit the activation of both JNK and p38 by the prevention of phosphorylation. We further used SP600125 as a specific inhibitor for the JNK-mediated pathway. PD98059, which inhibits MEK1/2, was used to impair downstream signaling of the Ras/RAF pathway. An AKT inhibitor and Y-27632 were used as AKT and ROCK pathway inhibitors, respectively. MG-132 is a proteasome inhibitor, which has been used previously to indirectly inhibit the NFκB pathway via the prevention of iκB degradation [32].

**Figure 4:**
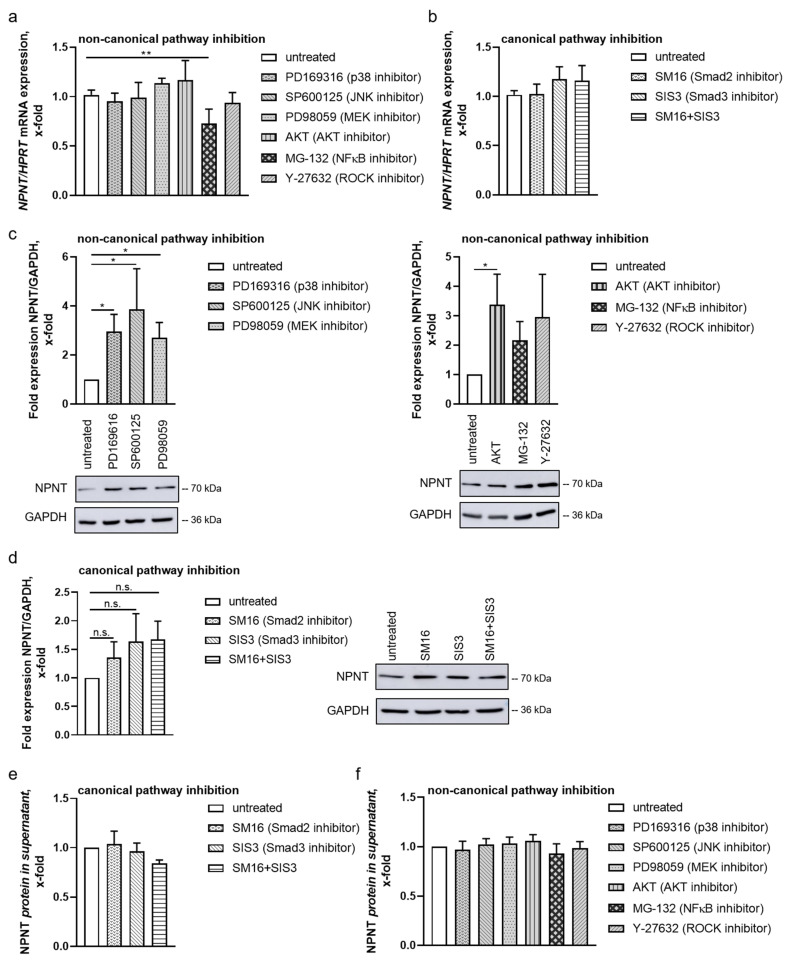
Non-canonical pathways predominantly mediate post-transcriptional regulation of NPNT under baseline conditions. (**a**,**b**) qPCR for NPNT mRNA in cultured human podocytes, untreated and after treatment with inhibitors of the non-canonical pathways (**a**), PD169316, SP600125, PD98059, AKT inhibitor, MG-132, and Y-27632, or with SM16 and/or SIS3 for canonical pathway inhibition (**b**). NPNT mRNA expression normalized to HPRT is given as the change compared to untreated cells. *n* = 6–7 independent experiments; ** *p* < 0.01. (**c**,**d**) NPNT protein expression in cultured human podocytes, untreated and after treatment with different inhibitors of the non-canonical (**c**) and the canonical pathways (**d**) as described in (**a**,**b**). NPNT protein expression, normalized to GAPDH, is given as the change compared to untreated cells. A representative Western blot result is shown for each treatment. *n* = 6–11 independent experiments; n.s. = not significant, * *p* < 0.05. (**e**,**f**) Human podocytes were treated as described in (**a**,**b**). NPNT protein expression was measured by ELISA in the cell culture supernatant and plotted as the change compared to untreated controls. *n* = 3 independent experiments.

Except for NFκB, separately blocking all arms of the non-canonical and canonical TGF-β pathways did not influence NPNT mRNA expression in podocytes (Figure 4a,b). In contrast, indirect inhibition of NFκB decreased NPNT mRNA. However, on protein level, inhibition of single molecules of the non-canonical TGF-β pathways increased NPNT expression in podocytes under baseline conditions (Figure 4c), which is in line with the hypothesis that NPNT is predominantly regulated post-transcriptionally at baseline.

In contrast, the inhibition of SMAD2 and SMAD3 only marginally upregulated NPNT protein expression in podocytes (Figure 4d). Thus, we conclude that, under baseline conditions, the non-canonical pathways have more impact on NPNT protein expression than the canonical pathway. The different findings in NPNT mRNA and protein suggest that the TGF-β-dependent downregulation of NPNT via the non-canonical pathways is mediated post-transcriptionally.

### 3.4. NPNT Protein Secretion Is Not Dependent on TGF-β Pathways

A potential mechanism that explains the observed differences in NPNT mRNA and protein expression is the repression of mRNA translation rates by TGF-β-induced miRs, TGF-β-mediated modulation of the NPNT protein half-life, or a TGF-β-dependent delay in NPNT protein secretion.

To examine the latter, we investigated NPNT protein levels in the supernatant of podocytes after pre-treatment with different TGF-β pathway inhibitors. The inhibition of SMAD2/3 had no significant effect on NPNT concentration in the cell culture supernatant of human podocytes (Figure 4e). Furthermore, treatment with different inhibitors of the non-canonical pathway also did not alter NPNT protein concentration in the cell culture supernatant of human podocytes (Figure 4f). This finding indicates that NPNT protein secretion is not influenced by the inhibition of TGF-β signaling.

### 3.5. TGF-β-Mediated Downregulation of NPNT Is Dependent on Non-Canonical Signaling Pathways in Podocytes

**Figure 5:**
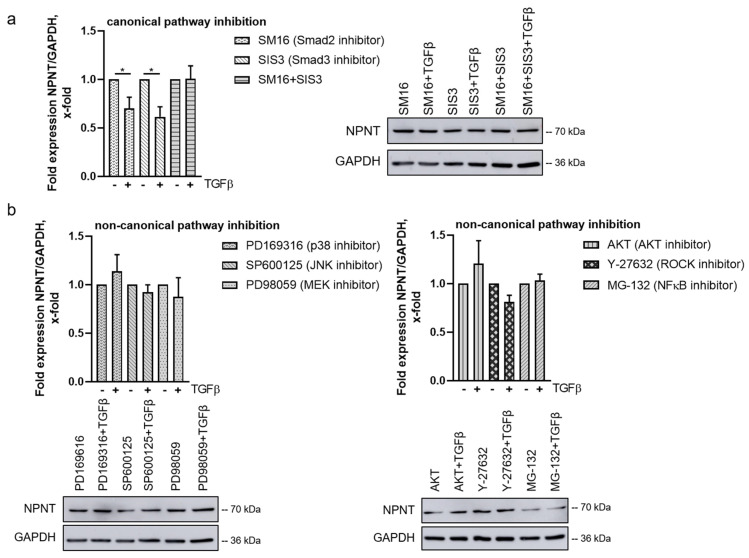
TGF-β-mediated downregulation of NPNT is dependent on the non-canonical signaling pathways in podocytes. (**a**,**b**) Western blot to determine NPNT protein expression in cultured human podocytes in the presence of (**a**) canonical (SM16 and SIS3) or (**b**) non-canonical (PD169316, SP600125, PD98059, AKT inhibitor, MG-132, and Y-27632) pathway inhibitors after stimulation with TGF-β. NPNT protein expression, normalized to GAPDH, is given as the change compared to stimulation with inhibitor without TGF-β. Representative Western blots are shown for each treatment. *n* = 5–6 independent experiments; * *p* < 0.05.

To further dissect the contributions of the canonical and the non-canonical TGF-β pathways on NPNT regulation in the presence of TGF-β, we analyzed human podocytes after treatment with the specific pathway inhibitors and stimulation with TGF-β. In the presence of the SMAD2- or SMAD3-inhibitors SM16 and SIS3, respectively, TGF-β was still able to downregulate NPNT protein expression in human podocytes (Figure 5a). However, the simultaneous inhibition of SMAD2 and SMAD3 impeded the effect of TGF-β on NPNT expression (Figure 5a). To investigate this further, we used murine wild type and Smad2/3 double-deficient podocytes in the same experimental setup. Our first observation was that Npnt protein levels between murine wild type and Smad2/3^-/-^ podocytes under baseline conditions cannot be directly compared, as the expression levels between the clones are different (data not shown). However, when we looked for NPNT expression after stimulation with TGF-β in murine podocytes of both genotypes, we observed that TGF-β decreased NPNT in the wild type podocytes as well as in the Smad2/3 double-deficient podocytes (Figure S2). Therefore, we conclude that the canonical TGF-β pathway is dispensable for NPNT regulation.

In contrast, incubation with different non-canonical pathway inhibitors (PD169316, SP600125, PD98059, AKT inhibitor, MG-132, as a surrogate inhibitor of NFkB, and Y-27632) prevented TGF-β from downregulating NPNT protein, suggesting that these pathways are important in mediating the TGF-β-mediated effect on NPNT and that they cannot fully compensate for each other (Figure 5b).

### 3.6. Non-Canonical TGF-β Pathways Regulate NPNT-Targeting miRs

**Figure 6:**
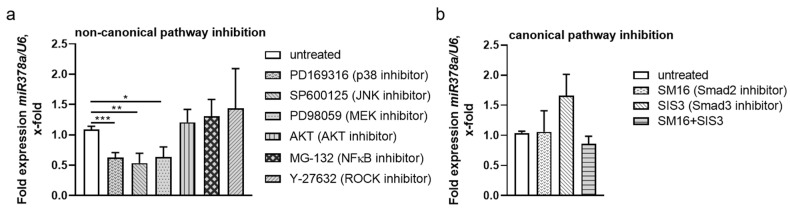
Post-transcriptional regulation of NPNT expression might be mediated by miR-378a-3p. (**a**,**b**): TaqMan PCR for miR-378a-3p detection in cultured human podocytes after treatment with inhibitors of the (**a**): non-canonical pathways (PD169316, SP600125, PD98059, AKT inhibitor, MG-132, and Y-27632) and (**b**): canonical pathway inhibitors (SM16 and SIS3), plotted as the change as compared to untreated cells. *n* = 4–6 independent experiments. * *p* < 0.05, ** *p* < 0.01 *** *p* < 0.001.

Due to the disparate findings in NPNT mRNA and protein expression in the aforementioned experiments, we suspected that miRs, upregulated upon stimulation with TGF-β, fine-tuned the TGF-β effect on NPNT expression in a feedforward loop. In a previous miR-screening in different cultured human glomerular cells, which were either untreated or stimulated with TGF-β, we identified miR-378a-3p to be specifically up regulated in podocytes after stimulation with TGF-β. In addition, we could show its effects on NPNT expression in previous publications [7,10]. Analyzing miR expression in human podocytes pre-stimulated with inhibitors of the non-canonical pathways revealed a significant reduction of miR-378a-3p after blocking the MEK (PD98059), JNK (SP600125), and p38 (PD169316) routes of the non-canonical TGF-β pathways (Figure 6a). In contrast, incubation with canonical pathway inhibitors (SM16, SIS3, and in combination) did not significantly alter miR-378a-3p expression (Figure 6b). Interestingly, while AKT inhibition increased NPNT protein abundance, it did not decrease miR-378a-3p expression, which indicates a different regulatory mechanism. This data hints that the reduced NPNT protein expression after TGF-β stimulation is mediated post-transcriptionally by miR-378a-3p.

## 4. Discussion

NPNT is an ECM protein produced by podocytes and glomerular endothelial cells and deposited in the GBM. We previously reported that TGF-β as well as podocyte-derived miR-378a-3p downregulate NPNT expression in human podocytes [7,10]. miR-378a-3p was further upregulated after stimulation with TGF-β in cultured human podocytes.

Many signaling responses induced by TGF-β are SMAD-dependent, but TGF-β can also signal SMAD-independently via non-canonical pathways [14,15,16], This alternative signaling route was also observed in podocytes [15]. TGF-β has been shown to activate the MEK, JNK, and p38 pathways and also NF-kB, Rock, and PI3K/AKT-dependent signaling [29].

We investigated the effects of different non-canonical and SMAD-dependent TGF-β pathways on NPNT expression at baseline and after stimulation with TGF-β by incubating human podocytes with different pathway inhibitors.

We could show a downregulation of NPNT mRNA, as well as intracellular NPNT protein, in podocytes after TGF-β treatment, whereas NPNT protein secretion and the intracellular protein half-life was not significantly influenced by stimulation with TGF-β.

To inhibit intracellular TGF-β signaling at the level of the TGF-β receptor, we incubated podocytes with a TGF-βR1 inhibitor. The TGF-βR1 inhibitor SD208 has been shown to be selective for TGF-βR1 without activity against other related kinases [18,28]. Compared to baseline, TGF-βR1 inhibitor increased NPNT protein expression. Interestingly, NPNT mRNA expression was unaltered after TGF-βR1 inhibition. We assume that this unexpected observation might be due to additional regulatory mechanisms, as we have already hypothesized post-transcriptional regulation of NPNT. In addition to that, a study by Lin et al. showed a direct inhibitory effect of TGF-βR1 on protein expression through phosphorylation (and thereby inhibition) of the elongation factor eEF1A1, which mediates recruitment of tRNA to the ribosome during protein synthesis [33]. Thus, inhibition of the kinase activity of TGF-βR1 impedes phosphorylation of eEF1A1 and thereby increases protein synthesis, as seen with NPNT.

We further analyzed NPNT mRNA and protein expression in the presence of different TGF-β pathway inhibitors under baseline conditions without additional TGF-β stimulation. Non-canonical pathway inhibitors that block ROCK, JNK, p38, AKT, or MEK signaling increased intracellular NPNT protein expression but had no effect on NPNT mRNA expression or protein secretion into the cell culture supernatant of human podocytes. Thus, under baseline conditions, these non-canonical pathways downregulate NPNT, probably by post-transcriptional mechanisms. Surrogate inhibition of the NFκB pathway via the proteasome inhibitor MG-132 decreased NPNT mRNA. Therefore, the NFκB pathway is presumably important in regulating NPNT on a transcriptional level and has opposing effects on NPNT expression compared to the other non-canonical TGF-β pathways. In line with these findings, NFκB is known to translocate into the nucleus after activation, by dissociation of IκBα, where it induces transcription [32,34]. However, as MG-132 is not specific for IκB degradation, further experiments are required to fully clarify the effects of the TGF-β-induced NFκB pathway on NPNT regulation.

Inhibition of the SMAD2/3-dependent canonical pathway only marginally increased NPNT protein expression in podocytes under baseline conditions, again with no significant changes in NPNT mRNA and NPNT protein in the supernatant. Thus, we suggest that the canonical pathway does not greatly impact NPNT regulation.

Since TGF-β is dysregulated in glomerular diseases [29], we next investigated NPNT expression after stimulation with TGF-β in the presence of different TGF-β pathway inhibitors. In the presence of a SMAD2 or SMAD3 inhibitor, TGF-β was still able to downregulate NPNT protein expression in human podocytes, confirming a minor role for this pathway in NPNT regulation. However, the simultaneous inhibition of SMAD2 and SMAD3 with the compounds used impeded the effect of TGF-β on NPNT expression in human podocytes. To investigate this further, we used murine Smad2/3 double-deficient podocytes. Analyzing Npnt protein expression after stimulation with TGF-β in both cell lines showed that TGF-β decreased Npnt protein in murine wild type podocytes, as well as in Smad2/3 double-deficient podocytes. Therefore, we concluded that the SMAD2/3 pathway is dispensable for NPNT regulation. In contrast, in the presence of different non-canonical pathway inhibitors, TGF-β was not able to downregulate NPNT, suggesting that these pathways are important in mediating the TGF-β effect on NPNT expression and that they cannot fully compensate for each other.

Previously, it has been reported that the functions of the specific factors within the TGF-β family vary and can even be opposing in different cells and at different developmental stages. Moreover, differential activation of TGF-β pathways is often highly context dependent yet plays important roles in a variety of cellular functions. It was suggested before that TGF-β-dependent NPNT expression is dominantly regulated through the ERK/JNK pathways in osteoblasts [35]. Furthermore, it was also discovered that SMAD-dependent pathways regulate NPNT expression in osteoblasts at baseline [36].

In addition to the context and cell-type-specific effects of the TGF-β pathways, the interaction between SMAD-dependent and SMAD-independent pathways were also described, making TGF-β-mediated intracellular pathway activations very complex.

TGF-β can either upregulate or downregulate gene expression. In a study by Ranganathan et al., 917 different genes were upregulated while 83 were downregulated by TGF-β in an immortalized lung epithelial cell-line (HPL1D). In lung adenocarcinoma cells, the number of genes regulated by TGF-β was even twice as high [37]. Interestingly, in line with our findings, the majority of TGF-β regulated genes were dependent on at least p38 MAP kinase, ERK kinase, or JNK kinase and their dependence was cell-type specific [13,14,38,39].

Some of the non-canonical pathways regulate SMAD activation, indicating the interaction of the different TGF-β pathways. The dual ability of TGF-β to activate SMADs and non-canonical pathways often results in cooperative effects. However, these pathways may also counteract each other. MAPK/Erk kinase 1 (MEKK1), which is upstream of p38 and JNK, can phosphorylate and activate SMADs [13]. A dual ability of TGF-β to activate SMADs and MAPK signaling was also described in epithelial to mesangial trans-differentiation [40,41]. Furthermore, TGF-β-induced activation of Erk and JNK pathways could phosphorylate SMADs in other cells [42,43]. Thus, the balance between the direct activation of SMADs and the MAPK pathways often defines cellular responses to TGF-β [13].

Finally, yet importantly, we looked for miR-dependent regulation of NPNT. TGF-β-mediated regulation of miRs might also be dependent on the baseline level of miR expression, as wells as positive or negative feedback loops, thereby resulting in a cell-type- and context-specific regulation. We recently reported the regulation of NPNT through miR-378a-3p [7,10]. Extending these findings, we have now observed that the inhibition of distinct parts of the non-canonical TGF-β pathways results in decreased miR-378a-3p expression, further stressing our hypothesis of post-transcriptional regulation of NPNT in response to TGF-β stimulation in podocytes. Interestingly, the AKT inhibitor strongly increased NPNT protein expression but was not able to decrease miR-378a-3p, suggesting a different mechanism. We believe that NPNT is also regulated by non-miR-378a-3p-mediated pathways after stimulation with TGF-**β,** as we could show in our previous study, where TGF-**β** was able to decrease NPNT in the presence of a miR-378a-3p inhibitor [10]. These mediators could be other TGF-**β**-inducible miRs, as we recently published in an miR screening of TGF-**β**-induced podocytic miRs [7] but also could be miR-independent pathways.

## 5. Conclusions

**Figure 7:**
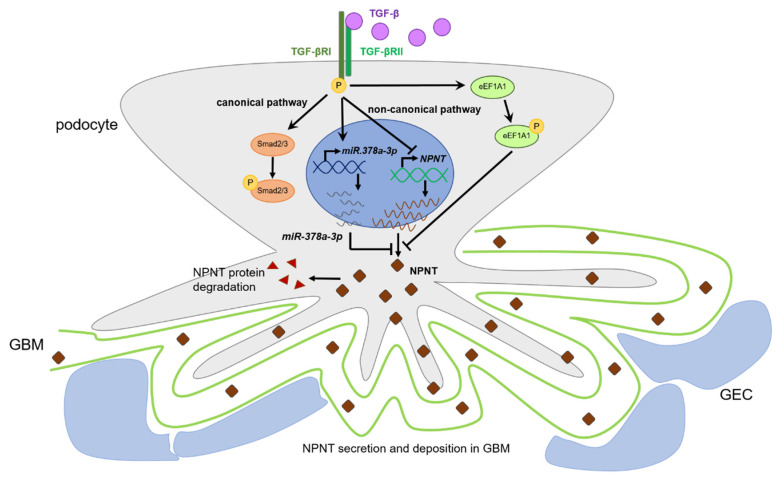
Proposed mechanism of podocyte NPNT regulation by TGF-β. We propose that podocyte NPNT expression is regulated primarily via the non-canonical TGF-β pathways. This regulation is mediated directly via inhibition of NPNT transcription and indirectly via miR-378a-3p. TGF-βRI-mediated phosphorylation of eukaryotic elongation factor 1A1 (eEF1A1) might further be involved in the regulation of protein translation [33]. Intracellular NPNT is either secreted and deposited into the glomerular basement membrane (GBM) or degraded. GEC = glomerular endothelial cell.

In summary, we propose a complex and fine-tuned network of NPNT regulation through different TGF-β pathways and TGF-β-dependent miRs in podocytes (Figure 7). We could show that podocyte NPNT expression is downregulated primarily via non-canonical TGF-β pathways. This regulation is mediated directly via the inhibition of NPNT transcription and indirectly via miR-378a-3p.

## Data Availability

The data presented in this study are available on request from the corresponding author.

## Supplementary Materials

The following are available online at https://www.mdpi.com/article/10.3390/cells11010149/s1, Figure S1: NPNT expression in podocyte cell fractions and protein half-life. Figure S2: NPNT expression in wild type podocytes as well as in the Smad2/3 double-deficient podocytes after stimulation with TGF-β.
