# Supplementary material for: A Tight Control of Non-Canonical TGF-β Pathways and MicroRNAs Downregulates Nephronectin in Podocytes
Source: Cells. 2022 Jan 3;11(1):149. doi: 10.3390/cells11010149 (PMC8750045; doi:10.3390/cells11010149)
Supplement: Supplementary file 1 [file cells-11-00149-s001.zip › cells-1508267-supplementary.pdf]

Supplementary material for:

A tight control of non-canonical TGF-β pathways and microRNAs downregulates nephronectin in podocytes

Nina Sopel [1], Alexandra Ohs [1], Mario Schiffer [1,2] and Janina Müller-Deile [1,3,*]

[1] Universitätsklinikum Erlangen, Department of Medicine 4 – Nephrology and Hypertension, 91054 Erlangen, Germany; nina.sopel@uk-erlangen.de; alexandra.ohs@uk-erlangen.de;
[2] Research Center On Rare Kidney Diseases (RECORD), Universitätsklinikum Erlangen, 91054 Erlangen, Germany, 91054 Erlangen, Germany; mario.schiffer@uk-erlangen.de
[3] Friedrich-Alexander-Universität Erlangen-Nürnberg, 91054 Erlangen, Germany; janina.mueller-deile@uk-erlangen.de
* Correspondence: janina.mueller-deile@uk-erlangen.de

Supplementary Figures:

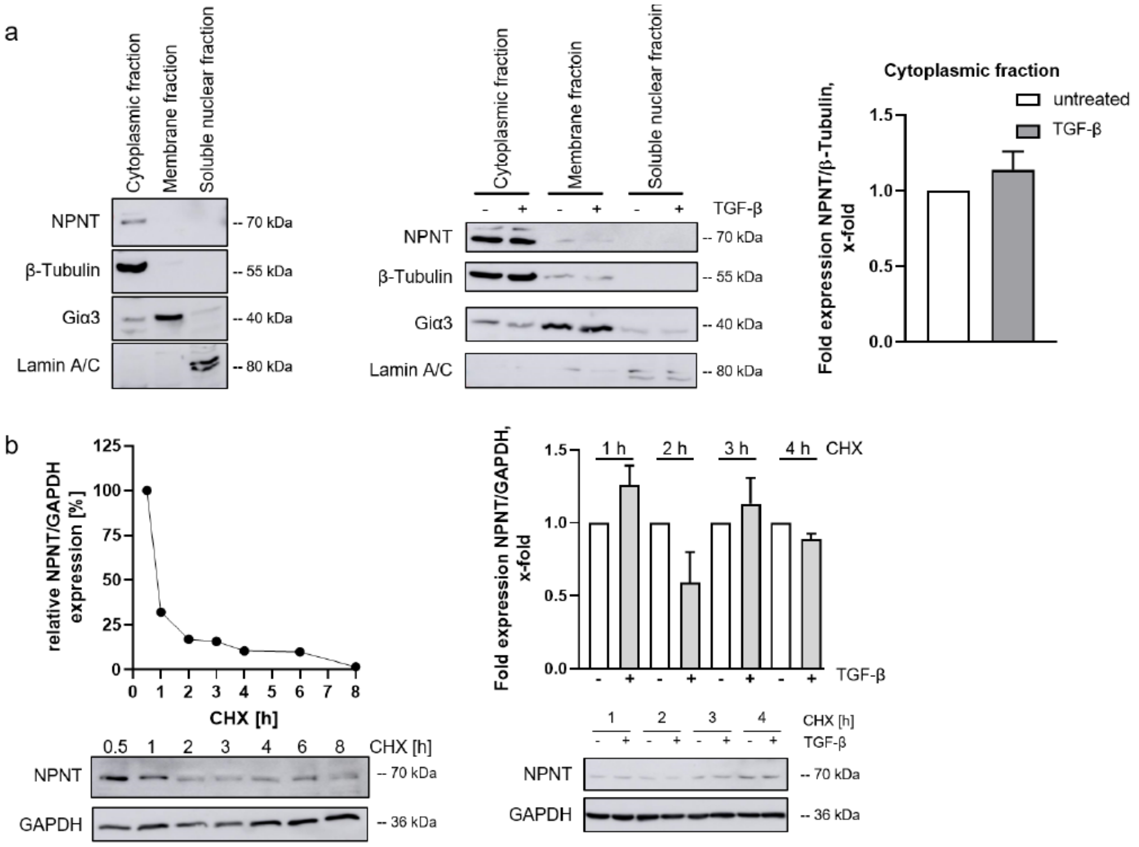

Figure S1. NPNT expression in podocyte cell fractions and protein half-life. a: NPNT protein expression in cytosol, membrane and nucleus of cultured human podocytes, as determined by markers for the respective fractions: β -tubulin, Giaα3 and lamin A/C. The left panel depicts a representative Western Blot of untreated human podocytes, the right panel a comparison of untreated and TGF-β treated human podocytes. A representative blot is shown. Densitometric analysis shows NPNT/β-Tubulin expression in the cytoplasmic fraction. n=2-3 independent experiments. b: Cycloheximide chase assay to determine baseline

protein turnover of NPNT in cultured human podocytes. Human podocytes were treated with cycloheximide (CHX) for the indicated time-points and NPNT protein was analyzed by Western Blot. Right panel: Protein turnover rate at each time point was illustrated as the percentage of NPNT/GAPDH at t = 0.5 of each time point. Left panel: Densitometric analysis of NPNT/GAPDH expression after 1 to 4 hours of CHX treatment of human podocytes in the presence or absence of TGF-β. n=2 independent experiments, representative experiments are shown.

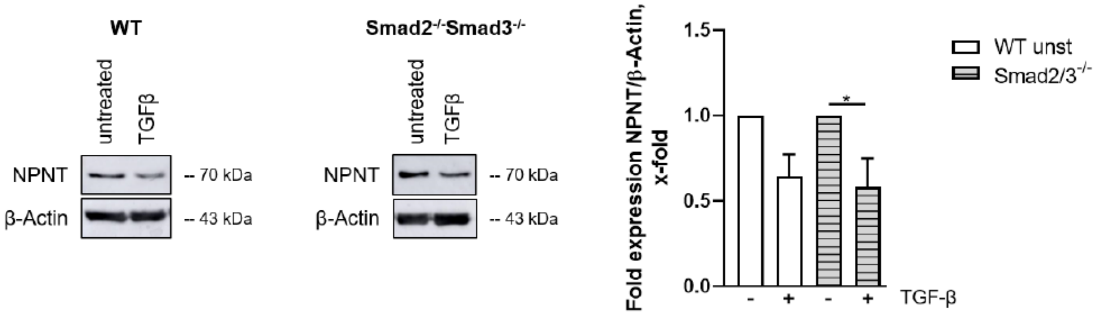

Figure S2. NPNT protein expression in murine wildtype (WT) and SMAD2- and SMAD3-deficient (Smad2/3-/-) podocytes. Western Blot analysis of NPNT protein expression in murine podocytes in the presence or absence of SMAD2/3 signaling and under baseline conditions or after treatment with TGF-β. NPNT expression is normalized to β-Actin and given as fold change compared to unstimulated cells, respectively. Representative western blots are shown for each treatment. n=3 independent experiments.
